# Philosophy of Life

**Published:** 1859-08

**Authors:** 


### PHILOSOPHY OF LIFE.

"Hardening the constitution" is a most pernicious phrase, when taken in connection with the popular notion as to how it is to be hardened; to wit, by exposures and severe toil. The true idea is to give it lastingness. To do this wisely, we must treat the constitution as we would a new garment, take care of it; and they who begin to do this soonest, and continue to do so with the greatest steadiness, live the longest. All severe exposures, all severe efforts, in proportion as they are violent or protracted, so far from hardening the constitution, sap its strength. All shocks impair the constitution; the first one not perceptibly, it may be, but it most certainly does its work. The first stroke upon a huge rock with a tiny hammer, may make no visible impression, but the same stroke repeated, will in time, break the mass asunder. The first stroke had an equal effect in reality with the last.

The hod carrier is a hard worker, but he does not live long. A mere out-door laborer, although in the pure open air all the time, does not live long. Nor is the farmer, with all the supposed advantages of a farming life, the longest liver among men. But hunters are, in spite of hunger, and privation, and the irregularities of their eating and sleeping, and activity and rest. Students, who have brains, who have sense enough to pay some attention to the laws of their being, have a better chance for a long life than out-door laborers or farmers; while the nobility of England, with their failure of labor, their drinking habits, with their customs of night dinners and midnight carousals, give more frequent examples, as almost any "steamer's news" will show, of extreme age, than either laborer or farmer, or wise student.

Without some first principles to guide him, the reader may regard this as an inextricable tangle; but with first principles, well founded, there is a beautiful and wise harmony, as there never fails, in any work of Him, who is the loving Father of us all.

To lay the foundation for a long life, both body and mind must practice industrious activities. The hod carrier works the body hard, the brain almost none; the power of one is used up, that of the other is not used at all, and he dies of some speedily fatal disease. The mere student exhausts the brain; the body is not worked at all, and he too dies early, with some acute malady. The farmer works his body hard; is in the open air all the time; eats plain food; retires early; rises with the sun; and indulges in no irregular habits; but his mind, beyond a certain routine, which soon becomes mechanical, as to prices, crops and weather, has no waking-up activities, and he too dies before his time, or vegetates in an asylum.

But the hunter, without the advantages of the regularity and abundance and comfort of a farmer's home, in spite of sleeping on the ground, and going whole days without food; in spite of winter's snows, and summer's suns, and the cold, raw rains of spring and fall, lives to the utmost verge of man's allotted time, and why? His bodily activities are steady, but they are moderate in the main, while the almost incessant look-out for game, and the multitudes of devices necessary to out-manœuvre the instincts of the animal creation, keep his wits alive, and they all become as keen and agile as his own restless and piercing eye.

The agencies of long life to the nobility of Great Britain, are their love of travel, and hunting, and the saddle in earlier years; while in later life, they avoid exposures and loss of rest and sleep and food; they, in the fullest sense of the phrase, "take things easy." They know that they are provided for, beyond a peradventure, and quietly and securely pass along the stream of life, until it empties into eternity's ocean.

As to great scholars and thinkers, such as Newton, of a past age, and Humboldt of the present, their love for study so took away their love of eating, that it was nearer a mechanical necessity than an animal delight; so they ate but little, and in such proportion had less need for exercise; while it is a physiological law, that mental labor increases our hold on life by its developing, enlarging (as all physicalities enlarge by exercise) the capacities of the brain.

Up to forty-five, the bodily constitution is knit, is built up, is consolidated by wise labors, if the mind also is kept in the exercise of healthful activities. The same hard labor after forty-five, so far from building up, destroys: but while that is the case, mental toil builds up the body, its effect is to increase the capability of living. Hence a man who works his body pretty hard and his mind rather more moderately up to forty-five, has done most towards securing a lasting constitution; and if then he begins to work the body less, and the mind more, he adds to that lastingness, and bids fairest to live to eighty or an hundred years. This article merits the mature reflection of every reader, for it is true literally that "out of it are the issues of life."

